# The health economic impact of disease management programs for COPD: a systematic literature review and meta-analysis

**DOI:** 10.1186/1471-2466-13-40

**Published:** 2013-07-03

**Authors:** Melinde RS Boland, Apostolos Tsiachristas, Annemarije L Kruis, Niels H Chavannes, Maureen PMH Rutten-van Mölken

**Affiliations:** 1 Institute for Medical Technology Assessment, Erasmus University Rotterdam, P.O. Box 1938, 3000, DR Rotterdam, Netherlands; 2 Department of Health Policy and Management, Erasmus University Rotterdam, Rotterdam, Netherlands; 3 Department of Public Health and Primary Care, Leiden University Medical Centre, Leiden, Netherlands

**Keywords:** Chronic obstructive pulmonary disease, Efficiency, Cost-effectiveness, Costs, Meta-analysis, Review, Integrated care, Disease management, COPD, Economic evaluation

## Abstract

**Background:**

There is insufficient evidence of the cost-effectiveness of Chronic Obstructive Pulmonary Disease (COPD) Disease Management (COPD-DM) programs. The aim of this review is to evaluate the economic impact of COPD-DM programs and investigate the relation between the impact on healthcare costs and health outcomes. We also investigated the impact of patient-, intervention, and study-characteristics.

**Methods:**

We conducted a systematic literature review to identify cost-effectiveness studies of COPD-DM. Where feasible, results were pooled using random-effects meta-analysis and explorative subgroup analyses were performed.

**Results:**

Sixteen papers describing 11 studies were included (7 randomized control trials (RCT), 2 pre-post, 2 case–control). Meta-analysis showed that COPD-DM led to hospitalization savings of €1060 (95% CI: €2040 to €80) per patient per year and savings in total healthcare utilization of €898 (95% CI: €1566 to €231) (excl. operating costs). In these health economic studies small but positive results on health outcomes were found, such as the St Georges Respiratory Questionnaire (SGRQ) score, which decreased with 1.7 points (95% CI: 0.5-2.9). There was great variability in DM interventions-, study- and patient-characteristics. There were indications that DM showed greater savings in studies with: severe COPD patients, patients with a history of exacerbations, RCT study design, high methodological quality, few different professions involved in the program, and study setting outside Europe.

**Conclusions:**

COPD-DM programs were found to have favourable effects on both health outcomes and costs, but there is considerable heterogeneity depending on patient-, intervention-, and study-characteristics.

## Background

Traditional healthcare for Chronic Obstructive Pulmonary Disease (COPD) focuses on pharmacotherapy to reduce symptoms and prevent exacerbations whereas patients are usually treated by a single healthcare professional, commonly the general practitioner or the respiratory physician. However, COPD is a multi-component disease with a wide range of comorbidities [1]. Essential determinants in improving health outcomes of COPD patients are behavioural changes in physical activity, diet and smoking. Thus, assessment and treatment of the airways alone is evidently insufficient in the care of COPD patients [2,3]. COPD requires an integrated, tailor-made approach. Such integrated approach mostly asks for a transformation in the healthcare organization from acute and reactive to proactive and planned healthcare. However, these behavioural and organizational changes require time and cannot be reached by implementing a single intervention. Instead, a set of organizational, professional-, and patient-oriented interventions is required for a successful change in organizational structure and processes as well as patient lifestyle and behaviour [4]. Disease management (DM) is such an approach and is seen as a solution to tackle the challenges posed by COPD.

Although DM programs are generally believed to be cost-effective, the available evidence is inconclusive. Several systematic reviews have evaluated the effects of COPD-DM [4-9]. However, they gave little insight into the economic consequences. Only the review of Steuten et al. [8], which searched studies between 1995 and 2007, included 3 studies which evaluated cost data. Since then, several studies focusing on DM and cost-effectiveness have been published. Furthermore, the review of De Bruin et al. [9], which searched for DM studies between 2007 and 2009, included 5 studies which evaluated cost data of COPD-DM programs, but did not include the studies before 2007 and after 2009. Furthermore, these two systematic reviews did not perform a meta-analysis on costs and effects. [4-9]In addition, little is known on the key elements of DM programs that are able to affect the outcomes and cost of COPD in a particular setting [5,10]. The great variation in DM interventions, study characteristics, patient characteristics, quality of study and a limited recognition of the impact of these differences on the outcomes are the reasons that evidence on cost-effectiveness of the DM programs provides limited support to decision makers [10]. The aim of this review is to evaluate the economic impact of COPD-DM programs and investigate the relation between the impact on healthcare costs and health outcomes. We also investigate whether this impact depends on intervention-, study-, and patient-characteristics.

## Methods

### Search strategy and selection criteria

A systematic electronic literature search for economic evaluations of COPD-DM was performed in Medline, the economic evaluation database of the UK National Health System (NHS-EED) and the EUROpean Network of Health Economic Evaluation Database (EURONHEED). The search was restricted to the English, German and Dutch language, but there were no restrictions to dates. All databases were searched on *21 July 2011*.

For the selection of the search terms, we firstly identified the key elements of DM. There are several definitions of DM available in the literature [11]. A short overview of definitions published in the last decade is shown in Additional file 1. Most definitions have eight elements in common that characterize DM, which are: 1) focusing on a target group of patients with a chronic condition, 2) multi-interventions developed for patient, healthcare provider and/or organization, 3) pro-active, planned healthcare, 4) evidence based/according to guideline, 5) self- management, 6) multidisciplinary team, 7) monitoring of performance and 8) supporting clinical information systems [11-18]. Furthermore, DM programs are often based on Wagner’s Chronic Care Model (CCM) [19], especially in Europe (EU). The CCM includes six interrelated components that are essential for improving chronic care. There are four elements at the micro level emphasizing interactions between patients, providers and community: 1) self-management, to empower and prepare patients to manage their disease (e.g. patient education, counselling to improve self-efficacy); 2) delivery system design, that assures the delivery of effective and efficient clinical and behavioural care (e.g. systematic and pro-active follow-up of patients); 3) decision support, to promote the use of evidence-based clinical care (e.g. electronic guidelines incorporated in information system); and 4) clinical information system, to assure access to timely, relevant data about patients (e.g. electronic patient record). One element at the meso level: 5) community, to link community and healthcare delivery. And one element at the macro level: 6) organizational support, to consider the policy and financing context. An indicated list with DM interventions grouped per CCM component is presented in Additional file 2. Overall, DM requires a change in routine care delivery for a prolonged period of time and DM programs often focus on the entire spectrum of severity of a disease and its complications, including often (secondary) prevention as well.

Besides the elements of DM, the search terms included descriptions of COPD, cost(s) and economic evaluation. The complete search strategy can be found in Additional file 3. Additional studies were sought by hand searching the reference list of reviews on economic evaluation of DM found in the literature search. The titles, keywords, abstracts and papers were screened to assess whether the study met the following inclusion criteria:

1. At least some of the patients had COPD and the results of the subgroup of COPD patients were presented separately;

2. The study included at least two DM interventions from the list presented in Additional file 2;

3. The study was an original empirical research paper excluding therefore, review, methodological and modelling studies;

4. The study reported both costs and effects;

5. The DM program had a minimum duration of 12 months (intensive + maintenance phase);

6. The comparator was usual care or no-intervention.

Potentially relevant studies retrieved from the electronic searches were identified by two reviewers (MB and AT) based on the predetermined inclusion criteria in a two-step procedure: 1) title, keywords, and abstract, 2) a brief screening of intervention, outcomes and costs. When disagreement of the two researchers could not be resolved by discussion, a third reviewer (MR) was consulted to reach consensus.

### Quality assessment, risk of bias and data analyses

We developed a check-list to assess the methodological quality of the studies based on the check-list of Drummond et al. [20] and the health technology assessment disease management instrument of Steuten et al. [21]. The former is used to assess the quality of economic evaluation studies in general and the latter is used to assess the methodological quality of DM evaluations specifically. The combined list assessed the strength and weaknesses of the studies on 7 key elements, each of which contains three or more items (see results section for the entire list) with a yes/no response option. The total quality score of a study is calculated as the sum of items with a positive assessment as a percentage of the number of applicable items. Hence, the maximum score is 100%.

We assessed the risk of bias of the individual studies according to the Cochrane Handbook for Systematic Reviews of Interventions [22] on five items of bias: (1) selection, (2) performance, (3) detection, (4) attrition, and (5) reporting. In order to prevent potential reporting bias, emails and telephone calls were made to the authors of the studies for additional information on the DM program, were necessary.

Given the likely heterogeneity between studies, we started with a descriptive analysis of the design, methods, quality, and results of the studies. A reviewer (MB) extracted data on study characteristics (sample size, setting, country, follow-up duration and study design), patient characteristics (age, gender, forced expiratory volume in one second as percentage of the predicted value (FEV_1_% pred), history of exacerbations and smoking status), type and number of interventions according to the CCM (i.e. self-management), type and number of healthcare provider(s) involved. Furthermore, the difference in cost per patient between the DM program and the comparator were reported according to the following categories: 1) DM development, implementation & operating costs, 2) direct costs of healthcare utilization, 3) direct costs of informal care, 4) direct non-medical costs borne by patients/families, and 5) costs of productivity loss. These were checked by a second reviewer (AT). The costs were inflated to 2010 [23] and were converted to Euros (€) by using Dutch purchaser power parities [24]. In addition, we reported the difference in outcomes which were grouped into the following categories: 1) care delivery process, 2) patient behaviour, 3) biomedical, physiological outcomes (e.g. lung function, body mass index (BMI)), 4) COPD-exacerbations, 5) health related quality of life and 6) mortality. Were possible, we have calculated relative risks for dichotomous outcomes and relative differences (RD), rate ratio (RR) or standardized mean differences (SMD) for continuous outcomes. To calculate a weighted average treatment effect, the data were pooled using a random-effects meta-analysis model based on the DerSimonian-Laird method [25] and the example of Linden and Adams [26]. Heterogeneity in the results was visually displayed using forest plots grouped into 1) intervention-, 2) study- and 3) patient characteristics.

## Results

### Description of studies

**Figure 1:**
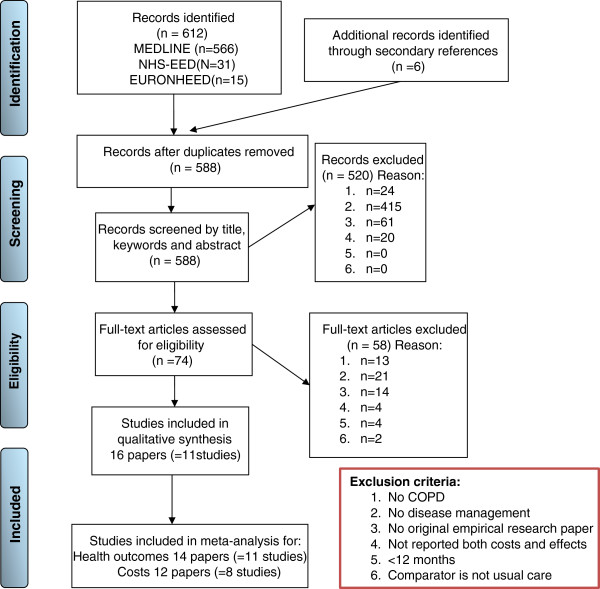
In- and exclusion of papers at various stages.

The literature search identified 612 potentially eligible papers and the screening of their references resulted in 6 additional papers. After the first step of selection (based on title, keywords and abstract) 544 papers were rejected. Examining the full text of the remaining papers led to the exclusion of 56 additional studies. The main reason for excluding were “no DM program” (n=436). Lastly, two additional papers were excluded because the comparator was not usual care or no-intervention. This resulted in the inclusion of 16 papers reporting 11 different studies. The reasons for excluding initially selected papers at various stages are presented in a PRISMA diagram [27] (Figure 1).

The selected studies included 7 randomized control trials (RCT), 2 pre-post and 2 case–control studies (Table 1). Six studies were conducted in Europe (the Netherlands (n=3), UK (n=1), France (n=1), Norway (n=1)) and five studies originated from non-European countries including USA (n=3), Canada (n=1), New Zealand (n=1). The duration of the DM program varied from 12 to 24 months. Some programs include an intensive phase followed by a maintenance phase; others do not make this distinction. In those that do, the minimum duration of the intensive phase was 3 weeks [28][29-31]. The sample size of the intervention group varied from n=16 [32] to n=524 [33], with a mean sample size of 160 (±168). The sample size of the control group varied from n=16 [32] to n=371 [34], with a mean sample size of 95 (±110). The average proportion of drop-out was 14% (±11).

**Table 1:** Study-, patient- and intervention characteristics

	**Study characteristics**							**Patient characteristics**										**Intervention characteristics**												
	**Country**^**a**^	**Follow-up (months)**	**Study design**^**b**^	**Sample size at baseline**^**c**^		**No of patients who completed study**^**c**^		**Mean age**		**Sex (% male)**		**FEV1% predicted**		**% with a history of ≥ 1 exacerbation in year prior to study**		**Smoking status (% smokers)**		Interventions categories according to CCM components*					Professions involved in delivering of the DM program**							
				**I**	**C**	**I**	**C**	**I**	**C**	**I**	**C**	**I**	**C**	**I**	**C**	**I**	**C**	**SMS**	**DEC**	**DSD**	**CIS**	**Total**	**RS**	**RN**	**GP**	**PHY**	**DIE**	**PHA**	**SW**	**Total**
[36]	NL	12	Pre-post	317	NA	222	NA	61	NA	56	NA	56	NA	NS	NS	40	NA	✓	✓	✓	✓	**4**	✓	✓	✓					**3**
[31]	NL	24	RCT	102	97	77	81	66	67	71	71	49	51	1.2	1.0	33	24	✓	✓	✓		**3**	✓	✓		✓	✓			**4**
[37]	FR	12	RCT	23	22	20	18	65	61	90	78	56	54	NS	NS	25	28	✓				**1**	NS	NS	NS	NS	NS	NS	NS	**NS**
[39]	USA	12	Case-control	94	47	NA	NA	NS	NS	NS	NS	NS	NS	NS	NS	NS	NS	✓	✓	✓		**3**	✓	✓	✓					**3**
[28]	NOR	12	RCT	31	31	26	27	57	58	48	52	52	55	NS	NS	39	39	✓	✓			**2**	✓	✓	✓	✓		✓		**5**
[38]	CAN	12	RCT	96	95	86	79	70	69	52	59	45	46	NS	NS	25	26	✓	✓	✓		**3**	✓	✓	✓					**3**
[29]	NL	12	RCT	127	121	122	114	65	65	85	84	56	58	1.4	1.3	28	26	✓				**1**	✓			✓				**2**
[30]	UK	24	RCT	61	61	55	49	70	70	49	49	43	49	100^e^	100	30	20	✓		✓		**2**		✓	✓					**2**
[34]	USA	12	RCT	372	371	336	323	69	71	98	98	36	38	100	100	22	23	✓	✓	✓		**3**	✓	✓		✓				**3**
[32]	NZ	12	Case-control	16	16	NA	NA	70	75	63	56	26	NS^d^	100	100	13	19	✓		✓		**2**	✓	✓	✓				✓	**4**
[33]	USA	12	Pre-post	524	NA	349	NA	64	NA	51	NA	NS	NA	NS	NA	NS	NA	✓	✓	✓		**3**	✓	✓	✓					**3**
Total (%)																		100	64	73	9		90	90	70	40	10	10	10	

^*a*^*NL* Netherlands, *FR* France, *USA* United States of America, *NOR* Norway, *CAN* Canada, *UK* United Kingdom, *NZ* New Zealand, ^*b*^*RCT* Randomized Control Trial, ^*c*^*I* Intervention, *C* comparison, ^*d*^*FEV*_*1*_ Control group 0.72 ± 0.22L, ^*e*^ in the last 4 years; *NS* Not stated, *NA* Not applicable, *SMS Self-management support, *DEC* Decision support, *DSD* Delivery system design, *CIS* Clinical information system, **RS Respiratory/chest specialist, *RN* Respiratory nurse, *GP* General practitioner, *PHY* Physiotherapist, *DIE* Dietician, *PHA* Pharmacist, *SW* Social worker.

The average age at baseline was 66(±4) for the intervention and 67(±6) for the control group (Table 1). The proportion of males varied more widely between studies with a mean proportion of males of 66% (±19) in the intervention group and 68% (±17) in the control group. The mean FEV_1_% of predicted was 47(±10) in the intervention group and 50(±7) in the control group, which indicates mild to moderate airflow obstruction according to the Global Initiative for Chronic Obstructive Lung Disease (GOLD) guidelines [35]. More than one fourth of the patients in the studies are smokers, with a mean proportion of 28(±10) in the intervention and 26(±6) in the control group. Determining the specific comorbidities of the patients was impossible. However, virtually all studies excluded patients with significant comorbidities. The study and patient characteristics per study are presented in Table 1.

Various DM interventions were evaluated in the studies (Table 1). All studies included interventions of the CCM component self-management support (SMS). Eight studies evaluated interventions of the component delivery system design (DSD), followed by decision support (DEC) (n=7) and clinical information system (CIS) (n=1). No study included interventions based on the CCM components organizational support (ORG) or community (COM). Two studies included multiple interventions in one CCM component, three studies included interventions covering two CCM components, five studies covered three components and one study [36] included interventions from 4 CCM components. Frequently applied interventions were (1) patient education on psychosocial effects of COPD (e.g. dealing with stress arising from living with a chronic disease, improving self-efficacy), knowledge of COPD and/or self-management skills (e.g. coping with breathlessness, exercise, encouragement of self-treatment), (2) stimulation of physical activity (e.g. fitness program in a small group), (3) changes in visits structure and organization (e.g. follow-up calls in response to exacerbation), (4) individual treatment plan, and (5) exacerbation management (e.g. patient training in recognizing early symptoms of exacerbation, discussion of individual causes of exacerbations, guidelines for self-treatment of exacerbations). The frequency of the interventions used in the included DM programs per CCM component can be found in Additional file 2.

The number of different professions involved in delivering of the DM program varied from two to five. One study did not report which healthcare providers were involved [37]. The most frequently involved healthcare providers of the DM programs were respiratory/chest specialist (RS) (90%), respiratory nurse (RN) (90%), general practitioner (GP) (70%) and physiotherapist (PHY) (40%). The intervention characteristics are shown in Table 1.

### The quality of studies and risk of bias

The quality score of the 11 selected studies varied between 29% and 80%. The mean score was 59% with a standard deviation of 16% (see Table 2). Most studies (82%) did not report detailed characteristics of institution(s) or region in which the intervention is implemented, e.g. size of the region and rural or urban environment. Only the setting of recruited institution(s) is known of all studies: 7 in a hospital setting [28-32,37,38], 1 in a primary care setting [36] and 3 in a combination of a hospital and primary care setting [33,34,39].

**Table 2:** Quality of study

**Characteristic**	**Type**	[36]	[31]	[37]	[39]	[28]	[38]	[29]	[30]	[34]	[32]	[33]	**%**
Study population	1. Clear description of in- and exclusion	✓	✓	✓	✓	✓	✓	✓	✓	✓	✓	-	91
	2. Clear description of drop-outs	-	✓	✓	-	✓	✓	✓	✓	✓	-	-	64
	3. The study population consist of an intervention and control group	-	✓	✓	✓	✓	✓	✓	✓	✓	✓	-	82
	4. Relevant baseline characteristics are comparable	NA	✓	✓	-	✓	✓	✓	✓	✓	✓	NA	89
Intervention	5. Random allocation	NA	✓	✓	-	✓	✓	✓	✓	✓	-	NA	78
	6. Clear description of type of intervention	✓	✓	-	✓	✓	✓	✓	✓	✓	✓	✓	91
	7. Clear description of the comparator	-	✓	✓	-	-	✓	-	-	-	-	-	27
	8. Detailed characteristics of institution(s)/region in which the intervention is implemented are described	✓	-	✓	-	-	-	-	-	-	-	-	18
	9. Co-interventions are avoided	✓	-	-	-	-	-	-	-	-	-	-	9
Measurement of all relevant cost categories	10. Inclusion of development /implementation /operating costs	-	-	-	✓	✓	✓	✓	✓	✓	✓	-	64
	11. Inclusion of healthcare utilization costs	✓	✓	✓	✓	✓	✓	✓	✓	✓	✓	✓	100
	12. Inclusion of direct non-medical and non-direct costs	-	✓	-	-	✓	-	✓	-	-	-	-	27
	13. Justification for omitting costs categories	-	-	-	-	NA	✓	NA	-	-	-	-	11
Measurement of all relevant outcome categories	14. Healthcare delivery process	✓	-	-	-	✓	-	-	-	-	-	-	18
	15. Patient behaviour	✓	✓	✓	-	-	-	✓	-	-	-	-	36
	16. Biomedical and physiological health outcomes	✓	✓	✓	✓	✓	✓	✓	✓	✓	✓	✓	100
	17. Health related quality of life and/or mortality and/or (quality) adjusted life years	✓	✓	✓	-	-	✓	✓	✓	✓	✓	✓	82
	18. Justification of omitting outcome categories	NA	-	✓	-	-	-	-	-	-	-	-	10
Measurement and valuation of data	19. Perspective explicitly mentioned	✓	✓	-	-	✓	✓	✓	-	✓	-	-	55
	20. The sources of resource utilization are described and justified	✓	✓	✓	-	✓	✓	✓	✓	✓	✓	✓	91
	21. The resource use and costs are reported separately	-	✓	-	✓	✓	✓	-	✓	✓	✓	✓	73
	22.The effects are measured in appropriate units	✓	✓	✓	✓	✓	✓	✓	✓	✓	✓	✓	100
	23. Data analysis is performed according intention-to-treat principle	✓	✓	-	-	-	✓	✓	-	✓	-	-	45
Presentation of data	24. Allowance made for uncertainty in the estimates of the costs	✓	✓	✓	-	✓	-	✓	-	✓	-	-	55
	25. Allowance made for uncertainty in the estimates of the effects	✓	✓	✓	✓	✓	-	✓	-	✓	-	-	64
	26. Incremental analysis of costs and effects are performed	-	✓	-	-	-	-	✓	-	-	-	-	36
Discussion of the study results	27. The results are interpreted adequate	✓	✓	✓	✓	✓	✓	✓	✓	✓	✓	✓	100
	28. The results are compared with other studies and allowances are made for potential differences in study methodology	✓	✓	✓	-	✓	✓	✓	✓	✓	✓	-	82
	29. The study discusses the generalizability of the results to other settings and patient groups	✓	✓	-	✓	-	✓	✓	-	✓	-	-	55
	30. The study discusses issues of implementation of the intervention	✓	✓	-	✓	-	✓	-	-	-	-	-	36
**Total quality of study (%)**		70	80	60	40	66	70	76	50	67	43	29	

*NA* not applicable.

Only one study [36] reported a plan to avoid contamination by other interventions and only three studies clearly provided details of the comparator [31,37,38]. Although all studies scored well on including intermediate and final health outcomes and costs of healthcare utilization, the lack of measurement of all relevant costs and outcome categories decreased the quality score of most studies.

Selection bias was likely in two studies [32,39] (see Additional file 4). One study [39] did not report patient-characteristics, which made it impossible to verify if the baseline characteristics were comparable. Both studies [32,39] did not randomly allocate patients. All studies had a high risk of performance, because blinding of the intervention for caregivers and patient is impossible. Although blinding of outcome assessors is possible, only 5 studies reported to have done so [28,31,34,37,38]. Four studies were at risk for attrition bias [32,33,36,39]. These four studies did not clearly describe the patients that dropped out from the study in a flow-chart or in the text. Moreover, one study [33] had a drop-out rate of 33%. Six studies were at risk of selective reporting, because they did not report statistical difference in costs and/or outcome [29,30,32-34,38].

### Results on costs

Various DM costs were included in the studies. Table 3 shows the results on difference in costs per patient (PP) between the DM program and the comparator. Of the 11 studies, 5 did not report statistical testing of the cost difference [29,30,32,33,38] and 1 study [34] reported only partly which costs differed significantly.

**Table 3:** Results on difference in costs per patient (€ 2010)

	**Development, implementation, operating**	**Healthcare utilization**							**Informal care**	**Direct-non medical costs**	**Productivity loss**	**Total**
		**Medication**	**Physician visits**	**Specialist visits**	**Other outpatient**	**ED visits**	**Hospitalization**	**Total healthcare utilization**				
[36]								-47				**-47**
[31]		668^1^	-12	-42	2038^1^		-424	2229^1^		-65	693	**2856**
[37]		-507*					1150	652				**652**
[39]	2007	79					-2098^2^	-2019*				**-13**
[28]	200	-182	-145*	-13			-708	-999		47*	-944	**-1689**
[38]#	2976		-22	-2		-158	-2448	-2630				**347**
[29]#	728	42	-6				-92	-56			280	**950**
[30]#	94		-79					-79				**15**
[34]#	545	13				-118	-936*	-1042				**-497**
[32]#	1850						-2004	-2004				**-154**
[33]#	712					-357	-1804	-2160				**-1448**

* Significant (p<0.05) ED visit= emergency department visit # no information of significance level available (n=5 studies), Dewan e.a. 2010 [34] reported only information on significance level of hospitalization and total costs - ^1^Significantly higher cost: diet nutrition, physiotherapist, dietician, respiratory nurse. ^2^Costs includes hospitalization, ED visits and Physician visits.

The total difference in costs between the COPD-DM program and the control group ranged from -€1689 [28] to €2856 [31], with a mean (±standard deviation (SD)) cost increase of €88 (±€1214). These total costs included the development, implementation and operating costs, where reported. Six of the eleven studies (55%) reported savings in total costs; however no study demonstrated a significant reduction of the total costs. On the other hand, no study demonstrated a significant increase of the total costs either.

The development, implementation and operating costs varied between €94 [34] and €2976 [38], with a mean (±SD) costs of €1139 (±€1022). The difference in healthcare costs PP varied between a cost reduction of €2672 [32] and a cost increase of €2229 [31]. Nine of the eleven studies (82%) reported healthcare costs savings, although the costs significantly decreased in only one of these studies [39]. The total healthcare costs were mainly driven by the hospitalization costs. All but one study [37] reported a reduction in hospitalization costs in favour of the DM programs. No study estimated the costs of informal care. Direct-non-medical costs borne by patients/family were included in two studies. One study [31] found a decrease in this type of costs of €65 and the other study [28] found a statistically significant increase in these costs of €47. The productivity costs were included in three studies: one study [28] showed a cost reduction of €944 and two studies [29,31] showed a cost increase of €693 and €280, respectively. These differences in productivity costs were not statistically significant.

**Figure 2:**
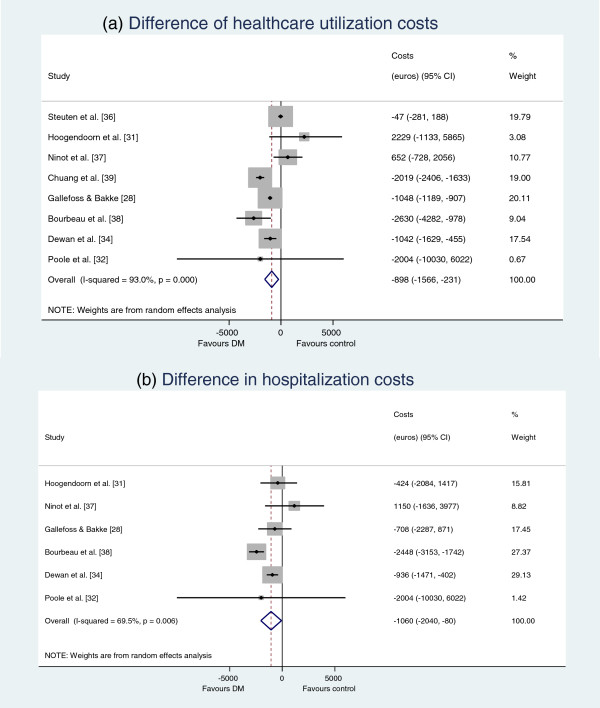
**Pooled results of the meta-analyses on costs.** Healthcare utilization costs **(a)** hospitalization costs **(b)**.

In total 11 and 9 studies reported total healthcare utilization and hospital costs, of which 8 and 6 studies provided enough data to be pooled in a meta-analysis, respectively. Figure 2a shows the results of the meta-analysis on healthcare utilization costs. COPD-DM programs were found to result in average healthcare savings of €898 PP (95% CI €231-€1566). The heterogeneity in healthcare costs across studies is large (I^2^= 93.0%). The pooled results from the 6 studies that included hospitalization costs demonstrated a reduction of €1060 PP (95% CI €80- €2040) (Figure 2b). However, the heterogeneity between studies in hospital costs is large (I^2^= 69.5%).

Table 4 shows the results of the meta-analysis of the COPD-DM programs by intervention-, study-, and patient-characteristics. Three intervention-characteristics were used to define subgroups for the meta-analysis: CCM components, number of different types of healthcare providers involved in the intervention and duration of the intervention. When data were pooled by the number of CCM components, the savings of programs covering 3 or more CCM components where greater than those of the programs covering 2 or less components. This difference was statistically significant for the hospital costs, but not for the total healthcare costs. Likewise, greater savings were found for studies with a long intervention duration (> 12 months), than for studies with a short intervention duration (< 12 months). These savings for studies with a long intervention duration were significant for the hospitalization costs, but not for the total healthcare costs. Subgroup analysis by number of professions involved in delivering of the DM program showed that interventions delivered by 2 or 3 disciplines of healthcare providers found significant savings in hospital costs as well as total healthcare costs but this was not found for interventions including 4 or more disciplines of healthcare providers.

**Table 4:** Pooled results of the meta-analysis of healthcare costs and hospitalization costs by subgroups

**Characteristics**	Subgroup*		**Healthcare utilization costs**		**Hospitalization costs**	
			**Study (N)**	**Mean difference (min-max)**	**Study (N)**	**Mean difference (min-max)**
**Intervention**	CCM	1-2	3	-428 (-1875 to 1018)	3	-311 (-1667 to 1045)
		3+	5	-1047 (-2230 to 137)	3	**-1378 (-2609 to -164)**
	Number of involved healthcare provider disciplines	2-3	4	**-1328 (-2554 to -101**)	2	**-1674 (-3155 to -192)**
		4+	3	-282 (-2510 to 1945)	3	-610 (-1770 to 550)
	Intervention duration (months)	0-12	2	-345 (-1986 to 1296)	2	-156 (-1820 to 1508)
		12+	6	-1066 (-2232 to 99)	4	**-1406 (-2566 to -246)**
**Study**	Design	RCT	5	**-866 (-1550 to -183)**		
		Non-RCT	3	-1074 (-2945 to 797)		
	Region	EU	4	-168 (-1043 to 706)	3	-323 (-1405 to 758)
		Non-EU	4	**-1731 (-2507 to -955)**	3	**-1681 (-3070 to -293)**
	Quality of study	0-60	3	-872 (-3253 to 1509)	2	806 (-1843 to 3456)
		60+	5	**-816 (-1543 to -89)**	4	**-1266 (-2283 to -250)**
**Patient**	Age	0-65	3	-307 (-1195 to 581)	2	-156 (-1820 to 1508)
		65+	4	-1128 (-2694 to 437)	4	**-1406 (-2566 to -246)**
	% male	0-60	4	**-929 (-1829 to -29)**	3	**-1790 (-3180 to -401)**
		60+	3	98 (-1568 to 1764)	3	**-738 (-1437 to -39)**
	GOLD	2	4	-168 (-1043 to 706)	3	-323 (-1405 to 758)
		3+	3	**-1558 (-2740 to -375)**	3	**-1681 (-3070 to -293)**
	Exacerbation	Yes	2	**-1047 (-1633 to -462)**	2	**-941 (-1474 to -407)**
		No**	6	**-850 (-1626 to -74)**	4	-920 (-2441 to 601**)**

*If subgroup assignment was impossible, the study was excluded in the meta-analyses.

** Having a history of one or more exacerbations was not stated in the inclusion criteria.

*CCM* Chronic care model, *RCT* Randomized control trials, *EU* European union, *GOLD* Global initiative for chronic obstructive lung disease.

Three study-characteristics were used to define subgroups: design, region and quality of study. Savings in hospital costs as well as total healthcare costs were found for non-EU countries but not for EU countries. COPD-DM programs with a non-RCT study design had on average greater healthcare savings than COPD-DM programs with a RCT study design. However, the savings were non-significant for non-RCT studies, whereas there were significant for RCT studies. Similarly, COPD-DM programs with a higher quality score found significant savings in total healthcare costs as well as hospital costs, whereas studies with a lower quality score did not.

Five patient-characteristics were used to define subgroups: age, percentage male, GOLD stage, a history of exacerbation as inclusion criteria and percentage smokers. Greater savings were found for COPD-DM programs with older patients, compared to younger patient. Finally, savings in healthcare costs as well as hospital costs were higher, when patients were more severely ill, i.e. had a higher GOLD stage and a history of exacerbations.

### Results on effects

Various DM effects were evaluated in the studies that reported costs. Of the 11 studies, 1 did not report statistical testing of the effects [32]. Changes in the process of care delivery were measured in one study, Steuten et al. [36], which demonstrated a significantly increased patient satisfaction with a RD of 0.13, indicating that the patient satisfaction increased by 13%. Changes in patients’ behaviour (e.g. physical activity, smoking behaviour) were measured in five studies. However, it was not possible to calculate the RR, RD or SMD due to a lack of information [21,29,31,37]. The only study [30] with complete information on change in patients’ behaviour showed positive results in favour of DM. In details, the RD in percentage of smokers was 0.01 and the self-use of antibiotics and steroids significantly increased with a RD in percentage of 17.92. Table 5 shows the results on effects of DM programs in RR, RD or SMD for the other outcomes.

**Table 5:** Results of effects of DM programs

	**Biomedical, physiological health outcomes and exacerbations**	**Health related quality of life**	**Relative Risk of mortality**
[36]	RD	RD	
	• Fev_1_% predicted= -0.02	• SGRQ= -0.03	
	• Fev_1_ reversibility= -0.27	• VAS= 0.03*	
	• Tiffeneau index= 0.02		
[31]	RR	SMD	• 1.01(0.30-3.37)
	• Hospitalization= 0.78 (0.69-0.89)	• SGRQ=-0.15*	
		• VAS= 1.01	
	• Exacerbations= 1.39 (1.10-1.74)	• EQ-5D= 0.17	
	SMD		
	• MRC= 0.58*		
	• 6MWD= 0.30		
	• Endurance time=0.37*		
	• Handgrip force= 0.24		
	• PI max= 0.29		
	• BMI= -1.22		
	• Fev_1_% predicted= -0.13		
[37]	SMD	SMD	
	• 6MWD= 0.99*	• SGRQ= 0.01	
	• Peak work rate=-0.88	• VAS=-0.07	
	• Peak Vo2= -0.06		
	• Energy= -0.30*		
	• Pain= 0.20		
	• Emotional reaction= -0.90*		
	• Sleep= 3.62		
	• Isolation= -0.40		
	• Physical mobility=0.18		
	• Voorrips total= 1.27*		
[39]	RR		
	• Hospitalization= 0.35 (0.29-0.43)		
	• ED visits= 0.39 (0.33-0.45)		
[28]	RR		
	• Days in hosp= 0.28 (0.24-0.32)		
	• Absenteeism from work= 0.05 (0.03-0.09)		
[38]#	RR	SMD	• 0.55 (0.19-1.58)
	• Hospitalization= 0.54(0.48-	• SGRQ= -0.29*	
	• 0.61)*		
	• Hospitalization 1 or more= 0.64(0.45-.91)*		
	• ED visits=0.64 (0.53-0.78)*		
	• ED visit 1 or more= 0.64(0.48-0.86)*		
	SMD		
	• Fev1=0.00		
	• FVC=0.00		
[29]#	SMD	SMD	• 0.95 (0.20-4.63)
	• 6MWD= -0.09	• SGRQ= -0.03	
[30]#	RR		• 0.50 (0.20-1.25)
	• Hospitalization= 1.20 (1.04-1.38)		due to COPD=0.13 (0.02-0.97)*
	• Hospitalization 1 or more= 1.08 (0.74-1.57)		
	• Exacerbation 1 or more= 1.00 (0.87-1.15)		
[34]#	RR		• 0.75 (0.50-1.13)
	• Hospitalization= 0.72 (0.65-0.79)*		
	• ED visits=0.73 (0.68-0.79)*		
[32]#	RR		• 0.33 (0.04-2.87)
	• Hospitalization= 1.72 (1.02-2.90)		
	• Hospitalization 1 or more = 1.08 (0.75-1.57)		
[33]#	RD	RD	
	• Hospitalization=-0.53	• SGRQ=-0.04	
	• ED visits=-0.66		
	• ICU admission=-0.57		
	• Absenteeism from work= -0.77		

* Significant (p<0.05) - *SGRQ* St. George's respiratory questionnaire, *VAS* Visual analogue scale, *FEV1%pred* % predicted forced expiratory volume in 1 second, *FEV1 Rev* Forced expiratory volume in 1 second reversibility, *MRC* Medical research council dyspnoea scale, *6MWD*= Six-minute walk distance, *EQ-5D* EuroQoL 5 dimensions, *PImax* Maximal inspiratory mouth pressure, *Peak VO2* Peak oxygen uptake mL^-1^ kg^-1^ min^-1^, *BMI* Body mass index, *ED visits* Emergency department visits, *FVC* Forced vital capacity, *Days in hosp*= Days in hospital, *ICU admission* Intensive care unit admission.

**Figure 3:**
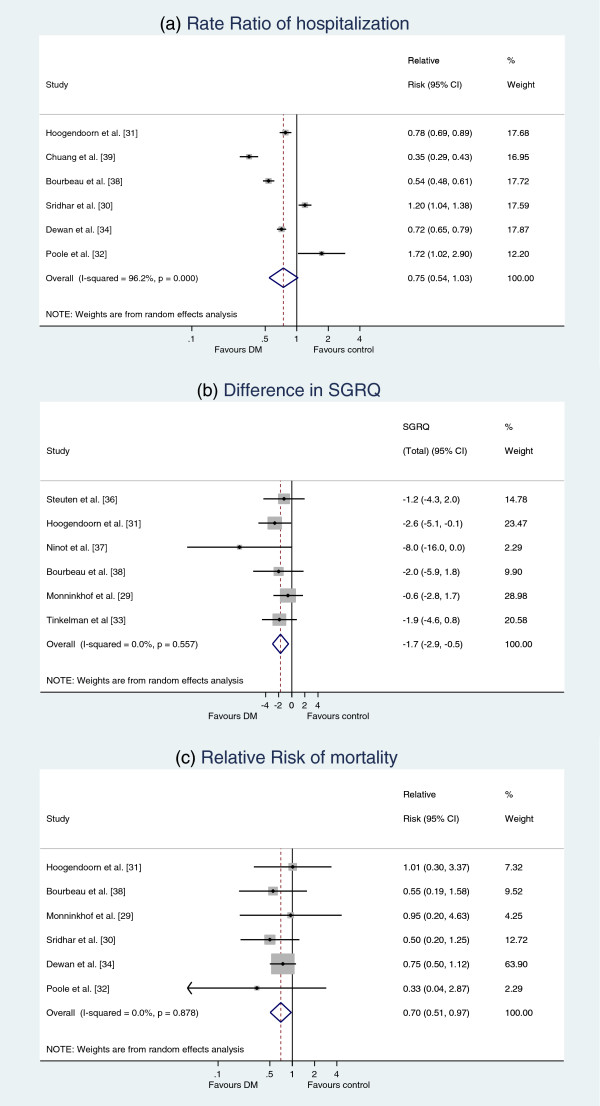
**Pooled results of the meta-analyses on health outcomes.** Rate Ratio of hospitalization **(a)** difference in SGRQ **(b)** Relative Risk of mortality **(c)**.

All studies measured changes in biomedical, physiological health outcomes or exacerbations. Hospitalizations as a proxy of severe COPD exacerbations were frequently reported. Two of the three studies that measured six-minute walk distance (6MWD) [31,37] showed an increased walking distance in the DM group compared to the usual care group, with the results being statistically significant in one of these two [37]. Three studies measured COPD exacerbations: two studies showed an increase of exacerbations [29,31], which was statistically significant in one study [31], but not in the other [30]. An exacerbation was defined differently across the three studies: *“an unscheduled need for healthcare, or need for steroid tablets, or antibiotics for worsening of their COPD*[30]*, a visit to the general practitioner or respiratory physician in combination with a prescription of antibiotics and/or prednisolone or a visit to the emergency department or day care of a hospital, which according to the patient, was related to a COPD exacerbation*[31]*, worsening of respiratory symptoms that required treatment with a short course of oral corticosteroids or antibiotics, as judged by the patient in the self-treatment group or by the study physician in the intervention and control groups*[29]*.”* Complete information on the RR of hospitalization was available for 6 of the 11 studies. Meta-analysis shows that DM programs decreased hospitalizations, but the RR was not significant (RR, 0.75, 95% CI, 0.54-1.03) (Figure 3a).

Changes in health related quality of life were described in 6 studies, which all used the SGRQ. Five of the six studies (83%) demonstrated an improved quality of life in favour of DM (Figure 3b), which was statistically significant in two studies [8,31,38]. The pooled results of the SGRQ showed a small significant reduction of the SGRQ in favour of DM (1.7 95% CI: 0.5 to 2.9). This reduction does not exceed the clinical relevant improvement of four points [40]. In addition to the SGRQ, three studies measured the health-related quality of life on a Visual Analogue Scale (VAS) and one study measured the EuroQol 5 dimensions (EQ-5D). Two studies [31,36] reported an increase in VAS in favour of DM and one study [37] showed a small decrease in VAS in favour of usual care. The one study [36] with significantly different results in the VAS showed a small increase (RD=0.03).

The number of patients that died during the study was described in 6 studies. Mortality never differed significantly between groups in individual studies, however the pooled Relative Risk showed a small significant reduction in all-cause mortality (0.70, 95% CI 0.51-0.97) (Figure 3c).

## Discussion

We systematically reviewed the impact of COPD-DM programs on both healthcare costs and health outcomes and highlighted the variations in intervention-, study-, and patient-characteristics.

The meta-analysis showed that DM led to average savings in healthcare costs of €898 PP (95% CI: €231 to €1566), hospitalization costs of €1060 PP (95% CI: €80 to €2040) and a decreased rate ratio of hospitalizations (0.75, 95% CI, 0.54-1.03). The costs of developing, implementing and operating the program were excluded from this estimate. Therefore, the results need to be interpreted with caution as the inclusion of all relevant costs could result in much lower cost savings, or even a total cost increase. Overall, six of the eleven studies reported savings on the total costs (including operating costs and non-medical costs), with a mean (±SD) costs increase of €88 (±1214). Interestingly, 6 studies did not report significance testing for the total costs and the remaining 5 studies did not demonstrate a significant reduction of the total costs.

The meta-analysis showed that the mean hospital costs savings (€1060 PP) were larger than the mean healthcare utilization savings (€898 PP). This is not caused by including different studies in the meta-analyses. It is also not unusual because a DM program initiates a more intensive treatment of the patient, often in primary or outpatient clinic care, in order to prevent hospital admissions or reduce the length of hospital stay. The more intense treatment leads to a cost increase, the prevention of admissions to cost savings, so overall savings in total healthcare costs are lower than savings in hospital costs.

Results of the quality assessment showed that the studies scored between 29 and 80, with a mean of 59. The studies that scored the lowest on our quality-instrument also had a substantial risk of bias [32,33,39]. Only 6 of the eleven studies (55%) scored more than 60 points. Studies with a lower quality score showed smaller savings in healthcare costs. This is related to the difference between RCTs and non-RCTs, where the first showed smaller but significant savings, whereas the latter showed greater but non-significant changes. The main problem in the methodological quality of the studies seems to be the lack of measuring all relevant costs and outcome categories, no clear description of the comparator or a description of the institution(s)/region in which the intervention was implemented. This complicates the interpretation of the study results. When trying to explain why results are different across studies, differences in patient characteristics are important. We found indications that DM led to greater savings in older patients, patients with a higher GOLD stage of airflow obstruction, and patients with a history of exacerbations. As these patients make more use of health care services, there is more room for cost savings.

Differences between intervention characteristics were also important. In line with previous reviews we found that patients who received 2 [6] or even 3 or more [4,8] interventions within different CCM components in DM programs for COPD had lower rates of hospitalizations. Consequently, savings in healthcare costs were also greater. Similarly, studies with a longer duration of follow-up showed greater reductions in hospital costs, because the relatively low frequency of hospital admissions requires a sufficiently long follow-up time to detect a reduction.

The aim of this review was to investigate the relation between the impact of COPD-DM programs on costs and their impact on health outcomes. Because costs and outcomes can only be related when they are obtained within the same study, we investigated the health outcomes that were reported in the papers reporting cost consequences of DM programs. Cost-effectiveness studies commonly relate costs to effects and calculate the addition costs per unit of additional effect (incremental cost-effectiveness ratio). However, there were only two studies reporting cost-effectiveness ratios [29,31]. Therefore, we had to review costs separately from the effects that were reported in the same studies.

There was a great variability in the type of outcome measures that were reported. Most DM programs led to changes in care delivery, as interventions to promote evidence based clinical care (e.g. education of healthcare provider, integration of specialist expertise in primary care) and interventions to promote effective, efficient care (e.g. systematic and pro-active follow-up of patients) were frequently provided as part of the DM program. Biomedical or physiological health outcomes and health related quality of life have shown small but positive changes in favour of DM. The quality of life results are in line with previous reviews. Niesink et al. [7] also demonstrated positive results of DM on quality of life in people with COPD. There was a lack of evidence on whether DM programs lead to changes in patient behaviour, although all studies provided interventions to empower and prepare patients to manage their disease (e.g. exacerbation management, individual treatment plan). This was also found in previous reviews [6,8].

Contrary to the positive biomedical or physiological outcomes, it is somewhat surprising that some studies found comparable [30] or even higher exacerbation rates for DM than for usual care [29,31]. Self-management training of the patients could have reduced the problem of under-reporting of exacerbations due to an improved ability of patients to recognize an exacerbation. DM programs could also have led to earlier detection of an exacerbation because of more frequent scheduled caregivers contacts [29,31].

Five previous systematic reviews investigated the effects of COPD-DM programs on health outcomes [4-8]. The results of COPD-DM programs on quality of life in these reviews were similar to our study. In more detail, 50% of the studies in the review of Niesink et al. [7], 67% of the studies in the review of Peytemann-Brideveaux [5] and 53% of the studies in the review of Steuten et al. [8] have shown statistically significant positive outcomes of COPD-DM on one or more domains of the quality of life instruments. The two studies that pooled data on the SGRQ demonstrated small but positive results in the DM group as compared to the control group. These results were statistically significant in the review by Lemmens et al. [4] (−2.52, 95% CI: -5.00, -0.05) and not statistically significant in the review by Adams et al. [6] (−0.25, 95% CI: -1.74, 1.24). Our pooled estimate of the improvement in SGRQ due to DM was −1.7 (95% CI: -2.9, -0.5). The effects of COPD-DM programs on mortality were estimated in two meta-analyses [5,6]. Both studies found lower mortality rates in the DM group, but the difference with the control group was not statistically significant. Our RR of 0.7 (95% CI: 0.51, 0.97) further supports the positive results of COPD-DM on all-cause mortality. Furthermore, the effect of COPD-DM on hospitalization was examined in two reviews. The odds ratio of hospitalization in the study of Lemmens [4] was 0.58 (95% CI: 0.40-0.83) and the relative risk in the study of Adams [6] was 0.79 (95% CI: 0.66-0.94) which are comparable to the RR of 0.75 (95% CI, 0.54-1.03) found in our study.

All studies in our review evaluated a mixed package of interventions. Determining the contribution of individual components of this package is impossible. Patient education on self-management was frequently included in the DM programs, most often in combination with changes in visit structure and stimulation of physical activity. Surprisingly few DM programs focused on structural smoking cessation support or nutritional therapy, i.e. only one DM program involved dieticians [31]. Overall, the categorization of the DM interventions based on the CCM components showed that all studies included interventions within the self-management support (SMS) component and none within the organizational support (ORG) or community (COM) components. However, these components are essential to support the structural implementation of a large DM program. It is likely that these studies did not explicitly address these components because of the relatively small-scale on which the programs were implemented or because the organizational, financial and societal conditions necessary to implement disease management were already in place.

COPD-DM programs have much in common with rehabilitation programs. We avoided the inclusion of these programs by excluding all studies that evaluated a short (usually 1–4 months), intensive, multidisciplinary program, in which exercise training (both muscle training and endurance training) was the main component, because DM aims to change the routine of care delivery for a prolonged period of time. However, stimulating physical exercise is an element of many DM programs and some programs e.g. [29,37] pay more attention to this than others. Also, some interventions start with a short intensive intervention phase, followed by a longer and less intensive maintenance phase e.g. [30,31]. The first part may resemble pulmonary rehabilitation whereas the latter part is clearly long-term DM. Because of this sliding scale it is sometimes difficult to make a clear distinction between a low-intensity community-based pulmonary rehabilitation program and an intensive DM program.

There are several limitations of this study. Firstly, most studies demonstrate a lack of data on other cost then the cost of healthcare utilization. The importance of these other costs is shown in the study of Hoogendoorn et al. [31] and Monninkhof et al. [29] where including productivity costs led to increased costs for the DM program. The study by Hoogendoorn et al. [31] also was the only study that included total healthcare costs irrespective of the reason of resource use whereas other studies included COPD-related healthcare costs. In addition, only two studies reported the incremental cost-effectiveness ratio of the DM program [29,31].

Secondly, we pooled the results of the DM programs despite the large heterogeneity. This heterogeneity is primarily due to the variety of different interventions included in a DM programs, the variety of study designs and the quality of the studies, and the variety of patient characteristics. We address this by conducting subgroup analysis by study-, intervention-, and patient characteristics. All across Europe, reimbursement decision makers face the difficult question whether or not to reimburse such programs on a wide scale. Theoretically, the potential savings of these DM programs are great, but the evidence for this is still quite sketchy. We believe we can give some guidance by bringing all this evidence together, discuss its quality, combine it into the best possible estimate of potential savings we can currently get, and try to identify patient- and intervention-characteristics that may contribute to greater savings.

Finally, the generally small proportion of COPD patients that was included in COPD-DM programs [41,42] may jeopardize the generalizability of the costs and effects of DM programs. The exclusion of COPD patients with multi-comorbidities will decrease the generalizability of the results to the entire population of COPD patients in which comorbidity is frequent. For instance, studies excluded patients suffering from any “serious” [29,37], “overwhelming” [32] or “significant” [30] comorbidities.

## Conclusions

This systematic review of the literature suggests that COPD-DM programs reduce hospital admissions and decrease hospital and total healthcare costs (excluding development and management costs of DM programs). They also improve health outcomes, including health-related quality of life. Results are however quite heterogeneous, varying by study-, intervention-, and disease-characteristics. Designers and managers of DM programs for chronic diseases can use this information to develop and target DM programs to maximise their cost-effectiveness. Future economic evaluations of DM programs should target a wider population of COPD-patients and be of higher methodological quality.

## Abbreviations

6MWD: Six-minute walk distance; BMI: Body mass index; C: Comparison; CAN: Canada; CCM: Chronic care model; CI: Confidence interval; CIS: Clinical information system; COPD: Chronic obstructive pulmonary disease; COPD-DM: Disease management for chronic obstructive pulmonary disease; DEC: Decision support; DIE: Dietician; DM: Disease management; DSD: Delivery system design; ED visits: Emergency department visits; EQ-5D: EuroQol 5 dimensions; EU: Europe; EURONHEED: The EUROpean network of health economic evaluation database; FEV1 Rev: Forced expiratory volume in 1 second reversibility; FEV1% pred: Forced expiratory volume in one second as percentage of the predicted value; FEV1: Forced expiratory volume in 1 second; FR: France; FVC: Forced vital capacity; GOLD: Global initiative for chronic obstructive lung disease; GP: General practitioner; Hosp: Hospital; I: Intervention; ICU: Intensive care unit; MRC: Medical research council dyspnoea scale; NA: Not applicable; NHS-EED: The economic evaluation database of the UK National Health System; NL: Netherlands; NOR: Norway; NS: Not stated; NZ: New Zealand; Peak VO2: Peak oxygen uptake mL-1 kg-1 min-1; PHA: Pharmacist; PHY: Physiotherapist; PImax: Maximal inspiratory mouth pressure; PP: Per patient; RCT: Randomized controlled trial; RD: Relative differences; RN: Respiratory nurse; RR: Rate ratio; RS: Respiratory/chest specialist; SD: Standard deviation; SGRQ: Saint Georges respiratory questionnaire; SMD: Standardized mean differences; SMS: Self-management support; SW: Social worker; UK: United Kingdom; USA: United States of America; VAS: Visual analogue scale.

## Competing interests

The author(s) declare that they have no competing interests.

## Authors’ contribution

MB was involved in the study design, literature search, review of the articles, extraction of the data, statistical analysis and writing of the manuscript. AT and MR contributed in the study design, literature search, review of the articles, interpretation of the data and revision of the manuscript. NC and AK participated in the study design, interpretation of the data and revision of the manuscript. All authors read and approved the final manuscript.

## Pre-publication history

The pre-publication history for this paper can be accessed here:

http://www.biomedcentral.com/1471-2466/13/40/prepub

## Supplementary Material

Additional file 1 **A short overview of Disease Management definitions from the last decade **[11,12,13,14,15,16,17,18] Click here for file

Additional file 2 **Interventions used in the included DM programs per CCM component **[19] Click here for file

Additional file 3 Search terms. Click here for file

Additional file 4 **Risk of bias **[28,29,30,31,32,33,34]**,**[36,37,38,39] Click here for file
