# Supplementary material for: The health economic impact of disease management programs for COPD: a systematic literature review and meta-analysis
Source: BMC Pulm Med. 2013 Jul 3;13:40. doi: 10.1186/1471-2466-13-40 (PMC3704961; doi:10.1186/1471-2466-13-40)
Supplement: Additional file 1 — A short overview of Disease Management definitions from the last decade [11,12,13,14,15,16,17,18] [file 1471-2466-13-40-S1.docx]

**Appendix 1. A short overview of Disease Management definitions from the last decade**

| Study | Definition |
| --- | --- |
| [12] | *“a combination of patient education, provider use of practice guidelines, appropriate consultation, and supplies of drugs and ancillary services”* |
| [13] | *“an organized, proactive, multi-component approach to healthcare delivery that involves all members of a population with a specific disease entity; care is focused on and integrated across i) the entire spectrum of the disease and its complications, ii) the prevention of comorbid conditions, and iii) the relevant aspects of the delivery system”* |
| [14] | *“an intervention designed to manage or prevent a chronic condition using a systematic approach to care and potentially employing multiple treatment modalities”* |
| [15] | *“a systematic and multidisciplinary approach to care for chronic conditions including a patient education component”* |
| [16] | *“multidisciplinary efforts to improve the quality and cost-effectiveness of care for selected patients suffering from chronic conditions”* |
| [11] | *“a group of coherent interventions designed to prevent or manage one or more chronic conditions using a systematic, multidisciplinary approach and potentially employing multiple treatment modalities”* |
| [17] | *“a group of coherent interventions, designed to prevent or manage one or more chronic conditions using a community wide, systematic and structured multidisciplinary approach potentially employing multiple treatment modalities. The goal of* chronic disease prevention and management *is to identify persons with one or more chronic conditions, to promote self-management by patients and to address the illness or conditions* according to disease severity and patient needs and based on the best available evidence*, maximizing clinical effectiveness and efficiency regardless of treatment settings) or typical reimbursement patterns. Routine process and outcome measurements should allow feedback to all those involved, as well as to adapt the programme”* |
| [18] | *“a system of coordinated healthcare interventions and communications for populations with conditions in which patient self-care effort are significant”* |
