# Supplementary material for: The health economic impact of disease management programs for COPD: a systematic literature review and meta-analysis
Source: BMC Pulm Med. 2013 Jul 3;13:40. doi: 10.1186/1471-2466-13-40 (PMC3704961; doi:10.1186/1471-2466-13-40)
Supplement: Additional file 2 — Interventions used in the included DM programs per CCM component [19] [file 1471-2466-13-40-S2.docx]

**Appendix 2: Interventions used in the included DM programs per CCM component**

|  | N | % |
| --- | --- | --- |
| **Organisational support** |  |  |
| Integrated financing |  |  |
| Specific subsidies for foreign population |  |  |
| Sustainable financing agreements with health insurers |  |  |
| **Community** |  |  |
| Cooperation with external community partners |  |  |
| Treatment and care pathways in outpatient and inpatient care |  |  |
| Involvement of patient groups and patient panels in care design |  |  |
| Discussion panel for community partners related to chronic care |  |  |
| Regional training course |  |  |
| **Self-management** |  |  |
| Individual treatment plan | 6 | 55 |
| Patient education on psychosocial effects of COPD (e.g. dealing with stress arising from living with a chronic disease), knowledge of COPD and/or self-management skills (e.g. coping with breathlessness, exercise, encouragement of self-treatment) | 11 | 100 |
| Smoking cessation counselling, tobacco weaning | 4 | 36 |
| Stimulation of physical activity (e.g. fitness program in a small group) | 8 | 73 |
| Nutritional therapy | 3 | 27 |
| Exacerbation management: patient training in recognize early symptoms of exacerbation, discussion of individual causes of exacerbations guidelines for self-treatment of exacerbations | 5 | 45 |
| Promotion of disease specific information |  |  |
| Support of self-management e.g. internet, email or sms, e-consultation, 24-h nursing helpline) | 1 | 9 |
| Tele-monitoring |  |  |
| Personal coaching |  |  |
| Motivational interviewing |  |  |
| Informational meetings |  |  |
| Mirror interviews |  |  |
| Education for patient and family | 1 | 9 |
| Regulatory skills |  |  |
| Proactive coping |  |  |
| **Decision support** |  |  |
| Evidence-based approach to care e.g. care standards, clinical pathways) |  |  |
| Uniform treatment protocol in outpatient and inpatient care |  |  |
| Training and independence of practice assistants |  |  |
| Professional education and training for care providers | 2 | 18 |
| Education of case manager | 1 | 9 |
| Audit and feedback to care providers | 1 | 9 |
| Reminders |  |  |
| Development and implementation of care protocols for immigrants |  |  |
| Structural participation in training sessions |  |  |
| Quality of Life questionnaire |  |  |
| Registration of process and outcome indicators |  |  |
| Qualitative evaluation of healthcare via focus-groups with patients |  |  |
| Periodic evaluation of DM interventions and feedback |  |  |
| Measurement of patient satisfaction |  |  |
| Multidisciplinary protocol | 1 | 9 |
| Encouragement of healthcare providers to adhere the guidelines | 1 | 9 |
| Integrate specialist expertise in primary care | 3 | 27 |
| **Delivery system design** |  |  |
| Multidisciplinary cooperation between outpatient and inpatient care |  |  |
| Development of health pathways and protocols |  |  |
| Substitution of inpatient with outpatient care |  |  |
| Specific plan for immigrant population |  |  |
| Meetings of different disciplines for exchanging knowledge/information |  |  |
| Monitoring of high-risk patients |  |  |
| Board of clients |  |  |
| Periodic discussion sessions between care professionals and patients |  |  |
| Stepped care method |  |  |
| Delegation of care from specialist to nurse/care practitioner | 1 | 9 |
| Changes in visits structure and organisation (e.g. regularly telephone call to address self-management items, follow-up calls in response to exacerbation) | 7 | 64 |
| Central coordination / case manager | 2 | 18 |
| **Clinical information systems** |  |  |
| Electronic Patient Records system (with/without patient portal) |  |  |
| Hospital Information System |  |  |
| Integrated Information System |  |  |
| Use of ICT for Internal and/or regional benchmarking |  |  |
| Systematic registration by every caregiver |  |  |
| Exchange of information between different care disciplines |  |  |
| Steering information to manage the programme | 1 | 9 |

Adjusted from: [19]
