# Supplementary material for: The health economic impact of disease management programs for COPD: a systematic literature review and meta-analysis
Source: BMC Pulm Med. 2013 Jul 3;13:40. doi: 10.1186/1471-2466-13-40 (PMC3704961; doi:10.1186/1471-2466-13-40)
Supplement: Additional file 3 — Search terms. [file 1471-2466-13-40-S3.docx]

**Appendix 3. Search terms**

The search strategy used in Medline was as follows:

1. “Pulmonary disease, chronic obstructive”[MeSH Terms]
2. “Pulmonary”[All Fields] AND “disease”[All Fields] AND “chronic”[All Fields] AND “obstructive”[All Fields]
3. “chronic obstructive pulmonary disease”[All Fields]
4. “COPD”[All Fields]
5. “Chronic obstructive airway disease”[All Fields]
6. “Chronic obstructive lung disease”[All Fields]
7. “Pulmonary emphysema”[All Fields]
8. “Chronic bronchitis”[All Fields]
9. “Chronic airflow obstruction”[All Fields]
10. “COAD”[All Fields]
11. OR 1-10
12. Cost-benefit analys*[MeSH Terms]
13. "Cost-benefit"[All Fields] AND analys*[All Fields]
14. "Cost"[All Fields] AND "benefit"[All Fields] AND analys*[All Fields]
15. “Cost”[All Fields]
16. “Economic”[All Fields] AND “evaluation”[All Fields]
17. Cost-effectiveness analys*[All Fields]
18. “Cost”[All Fields] AND “effectiveness”[All Fields] AND analys*[All Fields]
19. Health AND expenditure*
20. Healthcare AND expenditure*
21. “Health”[All Fields] AND “costs”[All Fields]
22. “Healthcare”[All Fields] AND “costs”[All Fields]
23. OR 12-22
24. “Disease management” [MeSH Terms]
25. “Disease management” [All Fields]
26. “Disease state management” [All Fields]
27. “Delivery of Health Care, Integrated”[MeSH]
28. “Case management” [All Fields]
29. “Comprehensive health care” [All Fields]
30. “Patient care management”[All Fields]
31. “Managed care”[All Fields]
32. “Managed care programs”[All Fields]
33. “Integrated”[All Fields] AND (“care”[All Fields] OR “health”[All Fields] OR “delivery ”[All Fields] OR system*)
34. “Patient-Centered Care”[All Fields]
35. (“Clinical”[All Fields] OR “critical”[All Fields]) AND pathway*)
36. “Care paths”[All Fields]
37. Guideline*
38. Practice guideline*
39. “Clinical protocol”[All Fields]
40. “Performance measurement”[All Fields]
41. (“Patient”[All Fields] OR “provider”[All Fields]) AND “Feedback” [All Fields]
42. (“Patient”[All Fields] OR “provider”[All Fields]) AND “Reminder” [All Fields]
43. (“Patient”[All Fields] OR “provider”[All Fields]) AND “Monitor”[All Fields]
44. “Reminder system” [All Fields]
45. “Decision support” [All Fields]
46. “Self-management”[All Fields]
47. “Self care” [All Fields]
48. (“Health”[All Fields] OR “patient”[All Fields] OR “provider”[All Fields]) AND “education” [All Fields]
49. “Health promotion” [All Fields]
50. “Community health planning” [All Fields]
51. “Planned health care”[All Fields]
52. “Pro-active”[All Fields]
53. “Continuity of patient care”[All Fields]
54. “Patient care planning” [All Fields]
55. Nursing care plan*
56. “Multiple interventions”[All Fields]
57. “Multiple”[All Fields] AND “interventions”[All Fields]
58. (Multidisciplin* OR interdisciplin*) AND (“care”[All Fields] OR “health”[All Fields] OR “delivery”[All Fields] OR “system”[All Fields])
59. “Central”[All Fields] AND “care”[All Fields] AND “giver”[All Fields]
60. “Patient care team”[All Fields]
61. “Patient tailored”[All Fields]
62. “Individual”[All Fields] AND “health plan”[All Fields]
63. “Patient care plan”[All Fields]
64. “Goals of care”[All Fields]
65. “Care goal”[All Fields]
66. “Pulmonary rehabilitation”[All Fields]
67. OR 24-65
68. English[lang] OR German[lang] OR Dutch[lang]
69. 11 AND 23 AND 67 AND 68

The search strategy used in NHS-EED and in Cochrane was as follows: "COPD in Title, Abstract or Keywords OR Chronic Obstructive Pulmonary Disease in Title, Abstract or Keywords AND Disease management in Title, Abstract or Keywords

The search strategy used in EURONHEED was as follows: “respiratory tract diseases” as disease and “COPD” as keyword.
