# Supplementary material for: The health economic impact of disease management programs for COPD: a systematic literature review and meta-analysis
Source: BMC Pulm Med. 2013 Jul 3;13:40. doi: 10.1186/1471-2466-13-40 (PMC3704961; doi:10.1186/1471-2466-13-40)
Supplement: Additional file 4 — Risk of bias [28,29,30,31,32,33,34],[36,37,38,39] [file 1471-2466-13-40-S4.docx]

|  | Risk of bias | | | | |
| --- | --- | --- | --- | --- | --- |
|  | Selection | Attrition | Performance | detection | Selective reporting |
| [36] | + | NA | NA | NA | + |
| [31] | + | + | - | + | + |
| [37] | + | + | - | + | + |
| [39] | - | - | - | - | + |
| [28] | + | + | - | + | + |
| [38] | + | + | - | + | - |
| [29] | + | + | - | - | - |
| [30] | + | + | - | - | - |
| [34] | + | + | - | + | - |
| [32] | - | - | - | - | - |
| [33] | - | NA | NA | NA | - |

+ = high risk of bias - = low risk of bias NA= not applicable
